# Supplementary material for: Learning curve and surgical outcome of robotic assisted colorectal surgery with ERAS program
Source: Sci Rep. 2022 Nov 29;12:20566. doi: 10.1038/s41598-022-24665-w (PMC9709162; doi:10.1038/s41598-022-24665-w)
Supplement: Supplementary file 2 — Supplementary Information 2. [file 41598_2022_24665_MOESM2_ESM.zip › RAL Raw data-Ñ[▒K/135/HS_ñw╝╨░O▒Kñσ.pdf]

正式摘要(電子簽章：000000233036325)<br>W52 / W52 ☐ 英文標題(注意:無英文姓名-請補病人基本檔!)	☐【讀取】憑證	

臺中榮民總醫院

出院病歷摘要

轉入號 : 轉出號 :

[redacted]

(8)流水編號	
(9)入院日期	110/12/17 (2021) 直腸外科 W52 - 122
(10)轉科日期	
(11)出院日期	110/12/24 (2021) 住院天數計 7 日

診斷

- 入院 : 1. S-colon tumor at15 cm FAV
  2. Left buccal ca s/p excision, adjuvant CCRT at Chung-Shan hospital in 2019
  3. Left buccal ca s/p exicion with left ALT free flap reconstruction in 2020/05, scar revision in 2021/01, pathology: superficially invasive SCC, WD
  4. CAD III, RCA s/p POBA+BMS stenting, LCX s/p POBA
  5. HCVD
  6. Hyperlipidemia
  7. Hypertension
  8. DM
- 出院 : 1. S-colon tumor at15 cm FAV
  s/p robotic AR on 2021/12/19
  Chronic colitis.
  2. Left buccal ca s/p excision, adjuvant CCRT at Chung-Shan hospital in 2019
  3. Left buccal ca s/p exicion with left ALT free flap reconstruction in 2020/05, scar revision in 2021/01, pathology: superficially invasive SCC, WD
  4. CAD III, RCA s/p POBA+BMS stenting, LCX s/p POBA
  5. HCVD
  6. Hyperlipidemia
  7. Hypertension
  8. DM

主訴

- tenesmus for about 3months

病史

- This 63 years old male patient has a medical history of
  1. Left buccal ca s/p excision, adjuvant CCRT at Chung-Shan hospital in 2019
  2. Left buccal ca s/p exicion with left ALT free flap reconstruction in 2020/05, scar revision in 2021/01, pathology: superficially invasive SCC, WD
  3. CAD III, RCA s/p POBA+BMS stenting, LCX s/p POBA
  4. HCVD
  5. Hyperlipidemia
  6. Hypertension 7. DM
  Due to buccal cancer, he had a regular visit to ENT OPD. He received PEF for f/u. The report shwoed lesion at colon. Thus, he was referd to our CRS OPD. At OPD, colonscopy was done and showed tumor at 15cm FAV. Biopsy was done. According to him, he received colonscopy at CSMU on 2018. No finding was noted that time. He denied bloody stool or BW loss. But he had a tenesmus for about 3months. Under the impression of S-colon tumor at 15cm FAV, the patient was admitted for Robot AR.

體檢發現

- OB 50
  COS: tumor 15 cm FAV
  - General appearance :
    - easy looking , conscious clear , without cardiopulmonary distress
    - Vital signs自動帶入date 體溫(BT) 脈搏(P) 呼吸(R) 血壓(BP) 身高(BH) 體重(BW) BMI

- o 20211217 36.1℃ 79次/分 18次/分 125/80mmHg 163.3cm 64.4kg 24.1
  - HEENT :
    - Eyes: no anemic conjunctiva, no icteric sclera, normal EOM
    - ENT: no otorrhea, no rhinorrhea, no erythematous mucosa, no injected tonsil, no deformity
  - Neck: supple, freely movable, trachea midline,no deformity, no engorged jugular vein , no palpable thyroid or lymph node enlargement
  - Chest:
    - Inspection: Grossly normal, symmetric and fully expansion, no accessory muscle used, no operation scar, no spider angiomata, no gynecomastia
    - Palpation: no palpable mass or nodule, no subcutaneous emphysema, no tenderness
    - Ausculation: clear breathing sound
  - Heart:
    - Percussion: normal heart size
    - Ausculation: regular heart beat, normal S1, S2, no gallop, no murmur
  - Abdomen:
    - Inspection: in
      - flat contour, symetrically, no scar, no stria, no dilated veins, no rashes
    - Ausculation: normoactive bowel sound, no abdominal bruits
    - palpation: soft, no muscle guarding or rigidity, no local tenderness, no rebounding pain, no palpable mass, no palpable pulsation
    - Percussion: no shifting dulllness, no hepatomegaly, no splenomagaly
  -

Peripheral pulsation :	CA	BA	RA	FA	PA	PTA	DPA
Right side :	++	++	++	++	++	++	++/+++
Left side :	++	++	++	++	++	++	++/+++

  - Genito-anus: no tenderness or palpable mass lesion or rectal shelf, normal external genitalia, normal anal tone, brown stool

CRS CONDITION

  - Colonoscope report: S-colon tumor at 15cm FAV
  - CEA: 2.27 ng/ml
  -
    - Lung

手術日期及方法

- 手術日期及方法20211218 1. Robotic (XI) assisted anterior resection
  2. Intraoperative colonoscopy : (+).
  3. High ligation (+) : At IMA root. Left colic artery and IMV were also ligated
  4. Take down from the splenic flexure : (-)
  5. Robotic endoGIA (45-4.3) X2 + EEA (29mm) were used
  6. Proximal cut end: 5 cm, distal cut end: 5 cm
  7. No drainage tube was inserted
  8. Indocyanine green agent was administered for checking the blood supply status of the remaining colon
  proximal colon time: 38 sec. distal rectum 42 sec

住院治療經過

- After admission, robotic AR was done on 2021/12/19. He start to try water on 12/21. Then, he ate bland I diet. Now he can tolerate Bland II deit. No obvious wound infection was noted. Under the stable condition, he was discharged today and arranged OPD f/u.

合併症

一般檢查

- 累積報告 :

DATE	TIME	ALB	TP	BIL.T	BIL.D	ALKP	AST	ALT	LDH	AMY	LIPASE	NH3	Ferritin	UA
1101218	1458	3.6		1.30				27						
1101220	0856			1.00	0.28		18	19						
1101223	0848	3.8		0.9				11						

DATE	TIME	WBC	RBC	HGB	HCT	MCV	PLT	BLST	NEUT	BAND	SEG	LYM	MON	EOS	BAS
1101218	1457	8890		14.8	44.4		135		89.9			8.0	1.9	0.1	0.1

1101220	0923	5920		13.9	42.3		110		72.8			18.9	7.4	0.7	0.2
1101223	0856	4130	4.58	13.7			122		62.5			22.8	8.7	5.8	0.2

DATE	TIME	NA	K	CL	BUN	CREAT	eGFR	CA	Mg	P	CRP	ESR	PCT	D-Dimer	LACTATE
1101218	1458	135	4.3	104	15	0.74	113.54	8.2							
1101220	0856	137	3.7	103	9	0.72		8.1							
1101223	0848	136	4.2	106	11	0.64	134.25	8.3							

- 放射線報告 :檢驗項目 **:CHEST AP VIEW (PORTABLE)** 報告日期 **:1101220**

  主　訴：
  Result:N
  Finding:
  > No cardiomegaly
  > Calcification of the aortic knob
  > Prominence of bilateral hila
  > Slightly increased hazy infiltrates in bilateral lungs
  > Mild degenerative change of T-spine
  > Post CVC placement
  > On EKG leads
  Impression:
  As above description.

  檢驗項目 **:CHEST PA VIEW** 報告日期 **:1101217**
  主　訴：
  Result:N
  Finding:
  1. The heart size is within normal limit with tortuous aorta
  2. Slight increasing infiltration over right lower lung field
  3. mild pleural thickening over right lung apex
  4. mild DJD changes over T-L spine
  Impression:
  As above description.

特殊檢查

病理報告

- 活體組織切片病理報告　手術日期：**110/12/14**　病理號：**S1041798**

診斷醫師:王任卿 報告日期:110/12/17
Pathologic diagnosis:
Sigmoid colon, 15 cm from anal verge, endoscopic biopsy --- Chronic colitis, mild, non-specific.
Gross description:
The specimen consists of 2 pieces of tan soft tissue, up to 0.5x0.2x0.2 cm. All for section.
Microscopic description:
Section shows colon mucosa with chronic inflammatory cells infiltration. Food debris seen.
Comment:
This specimen is superficial and may not be representative.
#T-59300_2 #M-43000_2 1 1340 000000

活體組織切片病理報告　手術日期：**110/12/18**　病理號：**S1042308**

診斷醫師:詹以吉 報告日期:110/12/27
Pathologic diagnosis:
1. Sigmoid colon, robot assisted colectomy --- a. Chronic colitis with submucosal edema. No tumor is found. b. Unremarkable mesocolic lymph nodes (0/17).
2. IMA root, dissection --- Unremarkable mesocolic lymph node (0/1).
3. Colon, cut end, distal, resection --- Unremarkable.
Ancillary study for diagnosis:
Recut done for section G and H.
Gross description:
The specimen consists of 1) a segment of colon, 10 cm in length and 5 cm in proximal cut end circumference, 7 cm in distal circumference, and mesocolic soft tissue. There is no tumor seen grossly. A stitch labeled the previous polypectomy site is seen, 3 cm away from the

- distal cut end. There are also 2 tiny mucosal buldging, up to 0.3x0.3 cm, noted 3 cm to the distal cut end. The both cut ends are free of tumor grossly. 2) IMA root, a piece of tan yellow soft tissue, 5x3.5x2 cm. 3) distal cut end, a piece of tan mucosa-covered soft tissue, 2.5x2.5x1 cm. Representative part for section: A) spcimen 2 B) specimen 3 C) proximal cut end D) distal cut end E) radial margin F) tiny mucosal buldging G-J) stitch site (inked by yellow dye) K-L) lymph nodes.
  #T-59470_2 #M-43000_2 0 1105 000000
  #T-C4560_2 #M-09183_2 0 1105 000000

其它

出院時情況

- 3.改門診治療

出院指示

藥名	劑量	劑量單位	用法	頻次	數量	數量單位	現況	天數	單位
Ultracet tab	1	TAB	PO	Q8HPRN	9	TAB	TH	3	天
(□)Takepron OD tab 30mg	1	TAB	PO	QDAC	3	TAB	TH	3	天
Mag. Oxide tab	2	TAB	PO	TID	18	TAB	TH	3	天
C.T.L. XR tab500mg	1	TAB	PO	QPM	3	TAB	TH	3	天
10mg Tulip tab	1	TAB	PO	QD	3	TAB	TH	3	天
Norvasc tab 5mg	1	TAB	PO	QD	3	TAB	TH	3	天

出院計劃

主治醫師 2222H 林俊余 / 住院醫師 2346C 陳怡豪

正式摘要(**電子簽章：000000233036325)**

W52 / W52

doctorRank:B
